# Genetic organization and mobile-element basis of β-lactam resistance in a polyclonal respiratory *Haemophilus influenzae* population

**DOI:** 10.3389/fmicb.2026.1945657

**Published:** 2026-09-29

**Authors:** Xiaomei Xu, Yupeng Luo, Juntao Tan, Na Li, Xiaoyu Hu, Dazhi Zhang

**Affiliations:** 1 Department of Infectious Diseases, Key Laboratory of Molecular Biology for Infectious Diseases (Ministry of Education), Institute for Viral Hepatitis, The Second Affiliated Hospital, Chongqing Medical University, Chongqing, China; 2 Department of Infectious Diseases, Affiliated Fifth People’s Hospital of Chengdu University of Traditional Chinese Medicine, Chengdu, China; 3 Department of Medical Laboratory Technology, Affiliated Fifth People’s Hospital of Chengdu University of Traditional Chinese Medicine, Chengdu, China; 4 College of Medical Informatics, Chongqing Medical University, Chongqing, China; 5 Department of Infectious Diseases, Hospital of Chengdu University of Traditional Chinese Medicine, Chengdu, Sichuan, China

**Keywords:** genome, *Haemophilus influenzae*, integrative conjugative elements, pangenome analysis, resistance

## Abstract

**Background:**

*Haemophilus influenzae* is an important respiratory colonizer and opportunistic pathogen. In the post-Hib vaccine era, non-type b and nontypeable strains have become increasingly associated with clinical disease, while β-lactam resistance complicates treatment and surveillance.

**Methods:**

To investigate the genetic basis and dissemination of β-lactam resistance, we performed comparative whole-genome analysis of 65 respiratory *H. influenzae* isolates from sputum and bronchial lavage fluid. The collection showed a highly polyclonal structure, including 28 known sequence types and 12 unassigned isolates.

**Results:**

Phylogenetic analysis demonstrated that β-lactamase-positive isolates were distributed across multiple lineages. Genotype-phenotype association analysis identified *blaTEM-1* as strongly associated with ampicillin resistance, whereas PBP3 substitutions including *ftsI_S357N*, *ftsI_S385T*, and *ftsI_L389F* were significantly linked to cefuroxime resistance. Several *blaTEM*-negative but ampicillin-resistant isolates carried similar compound *ftsI* mutation profiles, indicating that β-lactam susceptibility is shaped by both enzymatic resistance and PBP3 target modification. Pangenome analysis identified 4,223 gene clusters, comprising 1,224 core, 1,913 dispensable, and 1,086 private clusters. Presence/absence association analysis revealed that significant signals were concentrated in ampicillin resistance and formed a co-occurring dispensable-genome module containing *bla*, *tnpA*, *tnpR*, and multiple hypothetical proteins. This module mapped to a putative ICEHinR2866/ICEHin1056-like mobile genetic region carrying *blaTEM-1B*, conjugation-associated components, and macrolide resistance determinants.

**Conclusion:**

These findings suggest that β-lactam resistance in respiratory *H. influenzae* is driven by combined effects of β-lactamase production, composite PBP3 substitutions, and ICE-like mobile resistance platforms.

## Introduction

*Haemophilus influenzae* is a Gram-negative opportunistic pathogen highly adapted to the human respiratory tract. It can asymptomatically colonize the nasopharynx, but may also cause a broad spectrum of mucosal and invasive diseases, ranging from otitis media, sinusitis, bronchitis, and pneumonia to bacteremia. Based on capsular polysaccharide structure, *H. influenzae* can be classified into encapsulated serotypes a–f and non-encapsulated strains. The widespread use of the type b *H. influenzae* (Hib) conjugate vaccine has dramatically reduced the burden of Hib-associated invasive disease. With the decline of Hib disease, non-type b strains have become increasingly significant in respiratory tract infections and, in some cases, invasive infections, particularly among elderly individuals, patients with chronic underlying diseases, neonates, pregnant women, and immunocompromised populations (Langereis and de Jonge, 2015; Slack et al., 2021). This epidemiological shift has changed the central questions in *H. influenzae* research. Previous studies on the pathogenicity of *H. influenzae* primarily focused on capsule-mediated invasiveness, whereas the major clinical challenges in recent years increasingly come from respiratory isolates with complex genetic backgrounds and diverse resistance mechanisms. These isolates are capable of long-term persistence at respiratory sites, participate in recurrent or chronic infection processes, and play important roles in childhood otitis media and acute exacerbations of chronic obstructive pulmonary disease (Cerquetti and Giufrè, 2016; Ahearn et al., 2017; Wen et al., 2023).

Antimicrobial resistance further complicates the treatment of *H. influenzae* infections. β-lactams remain important agents for the treatment of respiratory *H. influenzae* infections, but their efficacy is challenged by both enzymatic and non-enzymatic resistance mechanisms. β-lactamase production is a major cause of ampicillin and amoxicillin resistance in *H. influenzae*, with blaTEM-1 and blaROB-1 representing common β-lactamase genes. At the same time, amino acid substitutions in penicillin-binding protein 3 (PBP3), encoded by ftsI, can reduce affinity for β-lactams and cause β-lactamase-negative ampicillin resistance or reduced susceptibility to some cephalosporins. Clinical studies have shown that β-lactam resistance in *H. influenzae* cannot be attributed to a single mechanism alone, but is shaped by β-lactamases, PBP3 remodeling, membrane permeability, efflux systems, and genetic background (Jakubu et al., 2021; Zhou et al., 2022). This mechanistic complexity limits the explanatory power of traditional molecular testing. β-lactamase detection identifies some aminopenicillin-resistant isolates but does not capture PBP3-mediated non-enzymatic resistance, whereas individual ftsI substitutions may not fully predict MIC values or categorical susceptibility. The increasing recognition of β-lactamase-negative ampicillin-resistant isolates and PBP3-altered strains in multiple regions indicates that *H. influenzae* resistance should be interpreted in terms of composite mutation profiles, population structure, and dispensable-genome context rather than single resistance markers alone (Kazuki et al., 2018; Kim et al., 2025).

How resistance determinants spread within bacterial populations is equally important for understanding the evolution of *H. influenzae* resistance. If resistance is driven primarily by the expansion of a dominant clone, control efforts focus on tracking clonal transmission. In contrast, recurrent occurrence of similar resistance modules in different genetic backgrounds would point to horizontal gene transfer and mobile genetic elements as major drivers of resistance distribution (Juhas et al., 2013). Integrative and conjugative elements (ICEs) integrate into the bacterial chromosome and can transfer between strains by conjugation, thereby acting as important vehicles for antimicrobial resistance genes. ICEHin1056 and related elements have been described in *H. influenzae*, and recent studies have shown that ICEs can carry resistance genes such as *blaTEM-1B*, *catA2*, *aac(6′)-Im*, *aph(2″)-Ib*, *mef(E)*, and *mel*. These findings suggest that ICEs can disseminate not only β-lactam resistance but also combined resistance modules involving β-lactams, macrolides, aminoglycosides, and chloramphenicol (Dimopoulou et al., 2007; Johannessen et al., 2022).

Although β-lactamase production mediated by blaTEM, non-enzymatic resistance mediated by ftsI/PBP3 substitutions, and ICE-associated resistance transfer have all been studied, how these mechanisms act together within respiratory *H. influenzae* populations from specific clinical settings remains insufficiently resolved. It remains important to determine whether β-lactam resistance in clinical respiratory isolates mainly reflects expansion of resistant clones or the distribution of resistance determinants across multiple genetic backgrounds. It is also unclear how blaTEM and composite ftsI/PBP3 substitution profiles jointly influence susceptibility phenotypes and whether resistance to commonly used β-lactams such as ampicillin is determined by isolated resistance genes or embedded within mobile genetic regions that link multiple resistance mechanisms. These questions are directly relevant to resistance evolution, molecular prediction, empirical therapy, regional surveillance, and antimicrobial stewardship. Whole-genome sequencing provides a framework for addressing these questions because it simultaneously resolves population structure, core-genome variation, pangenome composition, resistance genes, target-site mutations, and mobile genetic elements. Recent large-scale genomic studies have shown that H. influenzae, especially NTHi, has complex local and global population structures. One study including 4,474 isolates reported that 92.4% were NTHi and that most multidrug-resistant lineages also belonged to NTHi. These observations highlight the need to characterize *H. influenzae* population structure, resistance genomics, and clinical relevance at both local and broader scales (Slack et al., 2021).

Against this background, we performed comparative whole-genome analysis of 65 respiratory *H. influenzae* isolates recovered from sputum and bronchoalveolar lavage fluid (BALF). Rather than focusing on a single resistance gene or a single strain type, we analyzed susceptibility phenotypes within the context of phylogenetic structure and dispensable-genome variation, with particular attention to the relationship among *blaTEM*-mediated enzymatic resistance, *ftsI*/PBP3-mediated non-enzymatic resistance, and putative ICE-like mobile resistance-associated regions. This integrated framework aimed to clarify the multilayered genomic basis of β-lactam resistance in respiratory *H. influenzae* and to inform molecular resistance prediction, antimicrobial stewardship, and regional genomic surveillance.

## Results

### β-Lactamase-positive phenotypes are distributed across a polyclonal respiratory *H. influenzae* population

β-Lactam resistance in respiratory *H. influenzae* can accumulate in clinical populations through different routes. It may arise from local expansion of a resistant clone, or it may be driven by acquisition and distribution of resistance determinants across multiple genetic backgrounds. To distinguish between these possibilities, we first examined the population structure of the 65 respiratory isolates. Among them, 60 were recovered from sputum and 5 from BALF. Subsequently, the nitrocefin disk method was used to detect β-lactamase production, identifying 47 β-lactamase-positive and 18 β-lactamase-negative isolates, which indicated that β-lactamase positivity was common in this cohort (Supplementary Table 1). Sequencing and assembly revealed stable basic genomic features across the 65 isolates. Genome sizes ranged from 1.77 to 2.10 Mb, and GC content ranged from 37.85% to 38.32%. Assembly N50 values ranged from approximately 26 to 57 kb, maximum contig lengths ranged from 158 to 801 kb, and the overall proportion of ambiguous bases was low (Supplementary Table 2). Most assemblies showed Benchmarking Universal Single-Copy Orthologs (BUSCO) completeness of 98.3% to 99.1%, mainly represented by single-copy BUSCOs (Supplementary Table 3). Gene annotation identified approximately 1,657 to 2,018 CDSs, 56 to 70 tRNAs, 4 to 9 rRNAs, and one tmRNA in each genome. These consistent genomic features indicate that the isolate set had sufficient assembly completeness for population and comparative genomic analysis. MLST revealed substantial genetic diversity within the cohort. The 65 isolates were assigned to 28 known sequence types, whereas 12 isolates did not match known STs. No known ST dominated the collection. The most frequent types, ST103 and ST422, each contained only five isolates, and more than 16 STs were represented by a single isolate (Figure 1A, Supplementary Table 4). The minimum spanning tree further showed that the 65 isolates were distributed across 35 nodes. The 12 unassigned ST isolates were dispersed across 10 nodes rather than forming a single unassigned lineage (Figure 1B). Thus, the unassigned STs also represented multiple genetic backgrounds rather than local expansion of one unrecorded clone. Whole-genome SNP-based phylogenetic analysis supported this pattern at the whole-genome scale. The 65 isolates formed multiple branches rather than a single or a few tightly clustered clonal groups (Figure 1C). β-lactamase-positive isolates were not confined to a single phylogenetic branch. Instead, β-lactamase-positive and β-lactamase-negative isolates were interspersed across different branches (Figure 1C). This distribution is inconsistent with expansion of a single β-lactamase-positive lineage and indicates repeated occurrence of the phenotype across multiple genetic backgrounds.

**FIGURE 1:**
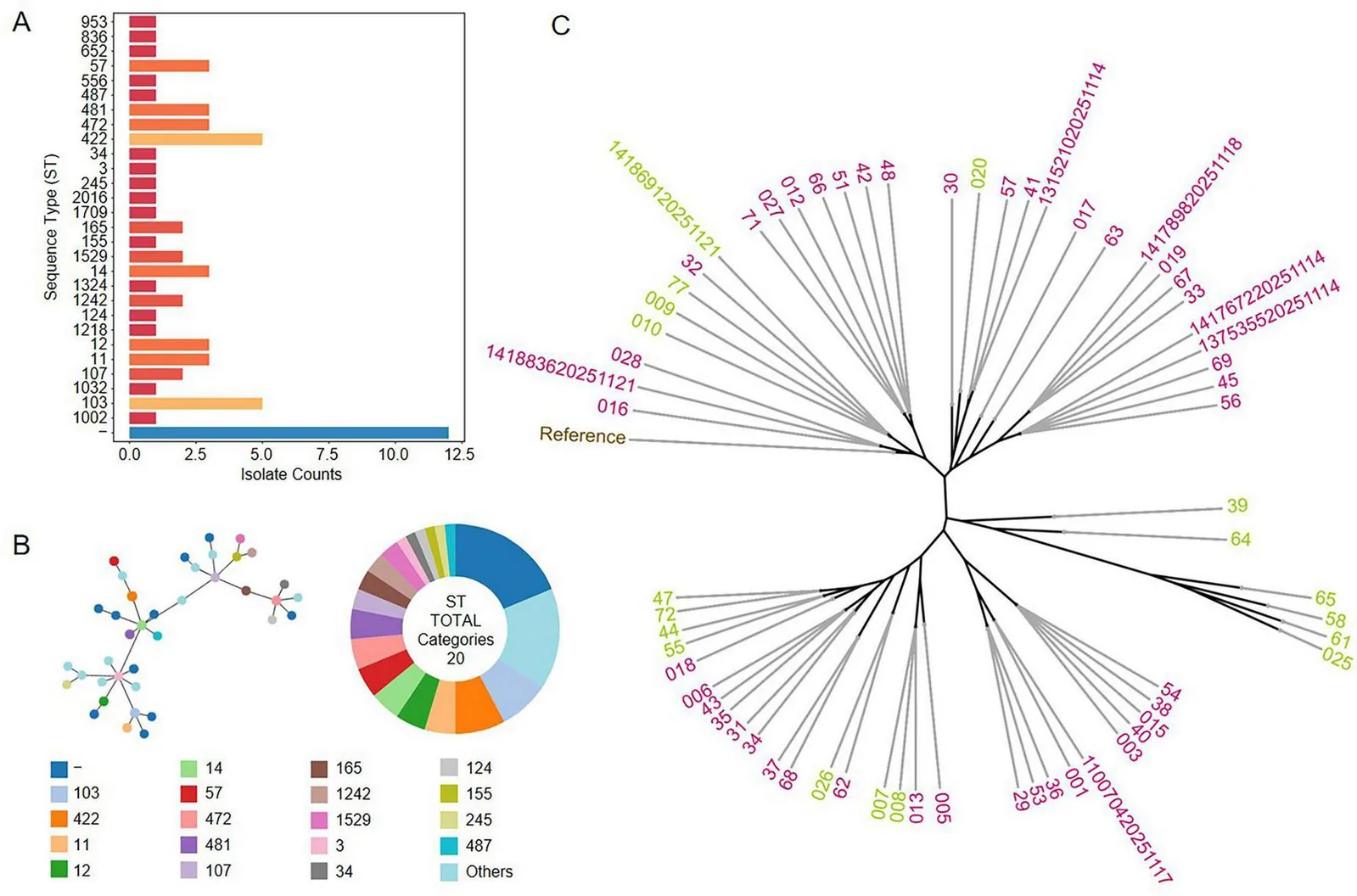
Genetic diversity and phylogenetic structure of respiratory *H. influenzae* isolates. **(A)** Distribution of sequence types (STs) among the 65 respiratory *H. influenzae* isolates based on multilocus sequence typing. The y axis indicates ST designations, with “-” denoting isolates that could not be assigned to a known ST in the PubMLST database. The x axis indicates the number of isolates assigned to each ST. Different colors represent different isolate counts, with STs having the same number of isolates shown in the same color. **(B)** Minimum spanning tree based on MLST profiles. Each node represents an ST, and genetically related STs are connected according to allelic similarity. Pie charts indicate the proportional composition of isolates within each ST node. Different colors represent different STs, with each ST assigned a distinct color. **(C)** Recombination-filtered genome-wide SNP-based phylogeny of the 65 isolates. Isolates are colored according to β-lactamase status, with purple indicating β-lactamase-positive isolates and green indicating β-lactamase-negative isolates.

### blaTEM and ftsI/PBP3 substitutions jointly explain β-lactam resistance phenotypes

β-Lactam resistance in *H. influenzae* is commonly driven by two mechanistic classes: β-lactamase-mediated enzymatic resistance and PBP3 target modification. To determine how these mechanisms contributed to resistance in our cohort, we characterized resistance genes and target-site mutations across all 65 isolates. Whole-genome screening identified 335 resistance-associated genes or mutation sites (Supplementary Table 5). Each isolate carried 2 to 10 determinants, with an average of 5.2 (Figure 2A, Supplementary Table 5). These determinants covered 17 resistance categories, indicating a complex resistance-associated genetic background in respiratory *H. influenzae* (Figure 2B). Determinants related to cell wall synthesis inhibitors were most prominent and primarily involved β-lactams and cephalosporins. Genes associated with efflux, quinolones, macrolides, tetracyclines, chloramphenicol, sulfonamides, and trimethoprim were also detected, indicating that the cohort did not carry only a single class of resistance markers. Focused analysis of β- lactam-, cephalosporin-, and efflux-related determinants identified 170 resistance-associated genes or mutation sites. The *hmrM* efflux pump gene was detected in all 65 isolates, whereas all isolates except isolate 39 carried at least one additional determinant. These determinants fell into 18 types, including the *hmrM* efflux pump gene, two *blaTEM* alleles, and 15 *ftsI*-encoded PBP3 substitutions (Figure 2C). This composition shows that the genomic basis of β-lactam resistance was not limited to β-lactamase genes and that multiple PBP3 substitutions constituted an important component of the resistance-associated genotype in this cohort. Among genotype–phenotype relationships, *blaTEM-1* was the most stable marker of ampicillin resistance. *blaTEM-1* showed strong concordance with ampicillin resistance, with a Kappa value of 0.593, an infinite odds ratio, and Fisher’s exact *P* < 0.001 (Table 1). All 41 isolates carrying *blaTEM-1* were resistant to ampicillin (Figure 2D). In contrast, resistant isolates in which *blaTEM-1* was not detected showed more complex genetic backgrounds. Two carried other *blaTEM* alleles, whereas the remaining four *blaTEM*-negative resistant isolates still tested positive for β-lactamase activity by the nitrocefin disk method. These atypical cases suggest that short-read assembly, sequence divergence, or gene-detection completeness may affect detection of some β-lactamase genes. The relationship between *blaTEM-1* and ampicillin-sulbactam resistance was weaker than that between *blaTEM-1* and ampicillin resistance. The Kappa value decreased to 0.462, and the odds ratio was 7.78 (Table 1). This difference is consistent with partial inhibition of β-lactamase activity by the β-lactamase inhibitor and indicates that the phenotypic explanatory power of the same resistance gene differs across drug combinations. No strong concordant marker was identified for amoxicillin-clavulanate, further suggesting that inhibitor-containing combinations weaken a simple one-gene-to-phenotype relationship. In contrast to the enzymatic resistance pattern observed for ampicillin, cefuroxime resistance was more strongly linked to PBP3 substitutions encoded by *ftsI*. The PBP3 S357N substitution showed the highest concordance with cefuroxime resistance, with a Kappa value of 0.512 and an infinite odds ratio (Table 1). PBP3 S385T was also significantly associated with cefuroxime resistance, with a Kappa value of 0.472 and an infinite odds ratio (Table 1; Figures 2E,F). PBP3 L389F also showed high concordance with cefuroxime resistance, with a Kappa value of 0.532 and an odds ratio of 12.19 (Table 1). These results indicate that specific PBP3 substitutions are important genetic correlates of cefuroxime resistance. The relationship between PBP3 substitutions and resistance phenotypes was not fully linear. All 33 cefuroxime-resistant isolates carried PBP3 S357N and S385T, yet some isolates carrying these substitutions remained susceptible (Figures 2E,F; Supplementary Figure 1). S357N and S385T therefore mark a PBP3 variant background associated with cefuroxime resistance but do not alone determine the final susceptibility category. PBP3 substitutions also contributed to ampicillin resistance. In addition to *blaTEM-1*, PBP3 substitutions M377I, S357N, and S385T showed concordance with ampicillin resistance (Table 1), suggesting that enzymatic resistance and PBP3 remodeling may jointly influence β-lactam susceptibility in some isolates. *blaTEM*-negative but ampicillin-resistant isolates further highlighted the importance of composite PBP3 substitution profiles. Four atypical resistant isolates carried similar *ftsI* mutation profiles encoding PBP3 substitutions S357N, S385T, M377I, R517H, and D350N, together with additional substitutions such as L389F and Y557H (Figure 2G). These composite profiles distinguished these isolates from susceptible strains and suggest that multiple PBP3 substitutions may help explain some β-lactamase-negative ampicillin-resistant phenotypes. Overall, β-lactam resistance in this cohort was shaped by both enzymatic and non-enzymatic mechanisms. *blaTEM-1* had strong explanatory power for ampicillin resistance, whereas composite PBP3 substitutions were more prominently associated with cefuroxime resistance and also contributed to some ampicillin-resistant phenotypes. The coexistence of strong genotype–phenotype associations and incomplete predictability indicates that single resistance markers do not cover the full genetic basis of β-lactam resistance in respiratory *H. influenzae*.

**FIGURE 2:**
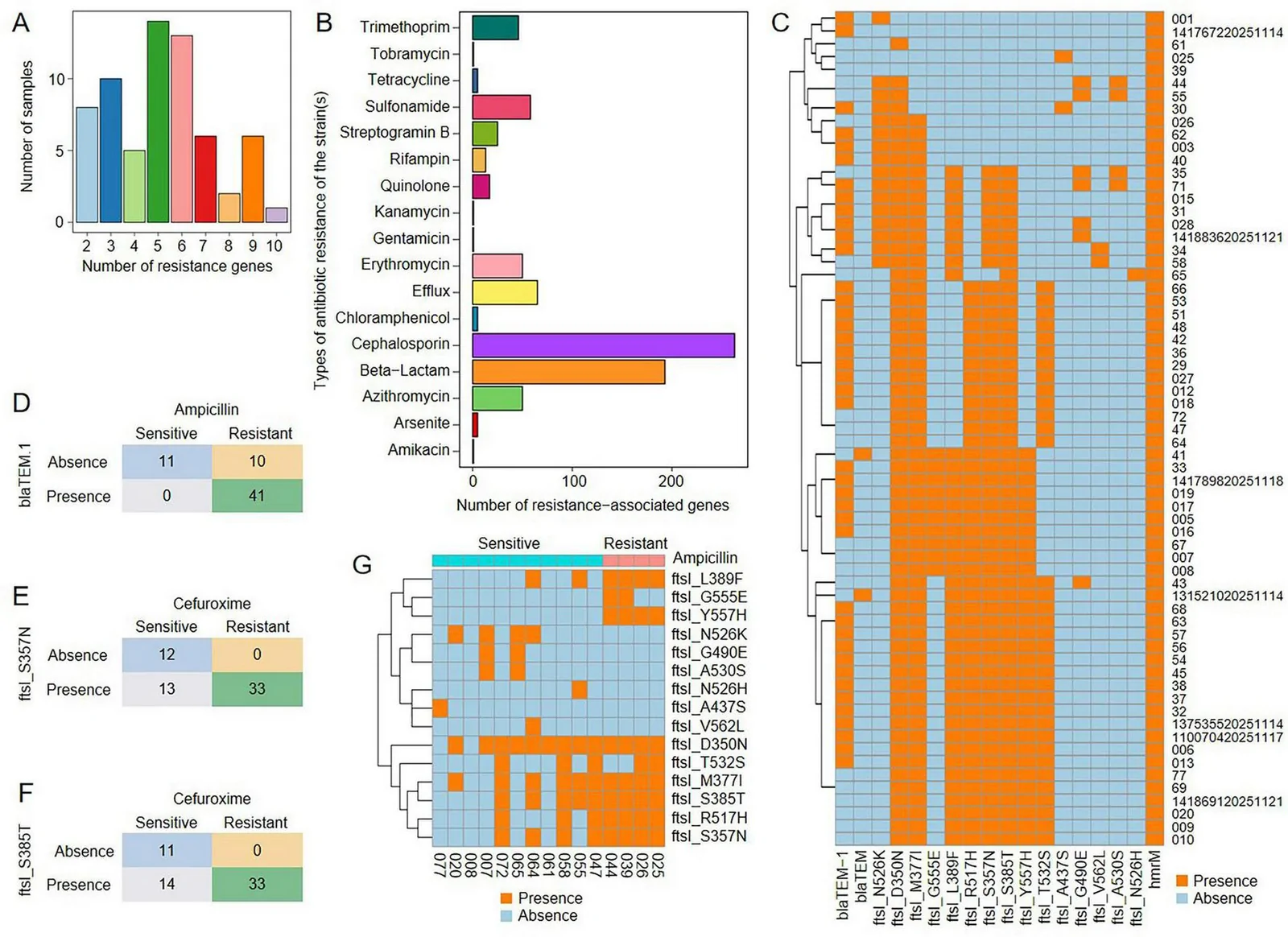
Antimicrobial resistance determinants and genotype–phenotype associations in respiratory *H. influenzae*. **(A)** Distribution of the number of antimicrobial resistance-associated determinants carried by each isolate. The x axis indicates the number of detected resistance-associated genes or mutation sites per isolate, and the y axis indicates the number of isolates. **(B)** Distribution of resistance-associated determinants across 17 predicted antimicrobial resistance categories. **(C)** Detection profile of β- lactam-, cephalosporin- and efflux-related resistance determinants across isolates, including resistance genes, *blaTEM* alleles and PBP3 amino acid substitutions encoded by *ftsI*. **(D)** Association between *blaTEM-1* and ampicillin resistance. **(E)** Association between the PBP3 S357N substitution encoded by *ftsI* and cefuroxime resistance. **(F)** Association between the PBP3 S385T substitution encoded by *ftsI* and cefuroxime resistance. **(G)** Heatmap showing combined PBP3 substitution profiles in 11 susceptible isolates and four atypical ampicillin-resistant isolates lacking detectable *blaTEM*. Blue indicates susceptible isolates and red indicates resistant isolates.

**TABLE 1:** Genotype–phenotype concordance between antimicrobial resistance determinants and β-lactam resistance phenotypes.

Gene	Phenotype	Kappa	Kappa_P	OR	OR_Lower	OR_Upper	Fisher_P	Sample_Size
*blaTEM-1*	Ampicillin	0.592641	3.23E-07	Inf	8.116154	Inf	6.94E-07	62
*blaTEM-1*	Ampicillin.Sulbactam	0.462254	0.000188	7.779385	2.220877	30.50856	0.000327	65
*ftsI_M377I*	Ampicillin	0.443114	0.000384	12.4155	1.896014	101.76	0.003025	62
*ftsI_L389F*	Cefuroxime	0.531677	3.85E-05	12.18764	2.97355	63.7019	6.00E-05	58
*ftsI_S357N*	Ampicillin	0.484193	0.000127	12.28382	2.350717	77.00483	0.000764	62
*ftsI_S357N*	Cefuroxime	0.51229	7.86E-06	Inf	5.73974	Inf	5.83E-06	58
*ftsI_S385T*	Ampicillin	0.413081	0.001125	8.530796	1.634015	49.17753	0.00423	62
*ftsI_S385T*	Cefuroxime	0.472042	2.31E-05	Inf	4.874932	Inf	1.96E-05	58

For Cohen’s Kappa analysis, isolates with an intermediate susceptibility phenotype were excluded, and only resistant and susceptible isolates were included in the binary comparison. Detailed susceptibility classifications for individual isolates are provided in Supplementary Table 1. Cohen’s Kappa analysis was used to evaluate concordance between selected resistance-associated genotypes and antimicrobial resistance phenotypes. The table lists resistance determinants, corresponding antimicrobial phenotypes, Kappa values, Kappa test *P* values, odds ratios, confidence intervals for odds ratios, Fisher’s exact test *P* values and sample sizes. *blaTEM*-1 showed the strongest concordance with ampicillin resistance, whereas PBP3 substitutions encoded by *ftsI*, including S357N, S385T, L389F and M377I, were associated with cefuroxime and/or ampicillin resistance.

### The dispensable genome reveals genetic heterogeneity and adaptive functional differences in respiratory *H. influenzae*

Resistance phenotypes in respiratory *H. influenzae* are not determined solely by a small number of resistance markers. They may also be shaped by broader differences in dispensable gene content. To examine genetic variation beyond the core genome, we constructed a pangenome for the 65 isolates. In total, 4,223 Roary-defined pangenome gene clusters were identified, including 1,224 core gene clusters, 1,913 dispensable gene clusters, and 1,086 private gene clusters (Figures 3A,B; Supplementary Figure 2). This structure shows that, although these isolates belong to the same species and have relatively stable genome sizes, their gene content differs substantially. The gene cluster presence/absence matrix further resolved how this variation was distributed across isolates. Core gene clusters were stably present in all isolates, whereas dispensable and private gene clusters constituted the main sources of inter-isolate differences. The number of dispensable gene clusters carried by individual isolates ranged from approximately 386 to 786, indicating uneven dispensable gene content across the cohort (Figure 3C). Private gene clusters were concentrated in a small number of isolates, especially isolates 19, 25, and 58, suggesting that some isolates carried more distinctive gene content or locally acquired genetic segments (Figure 3C).

**FIGURE 3:**
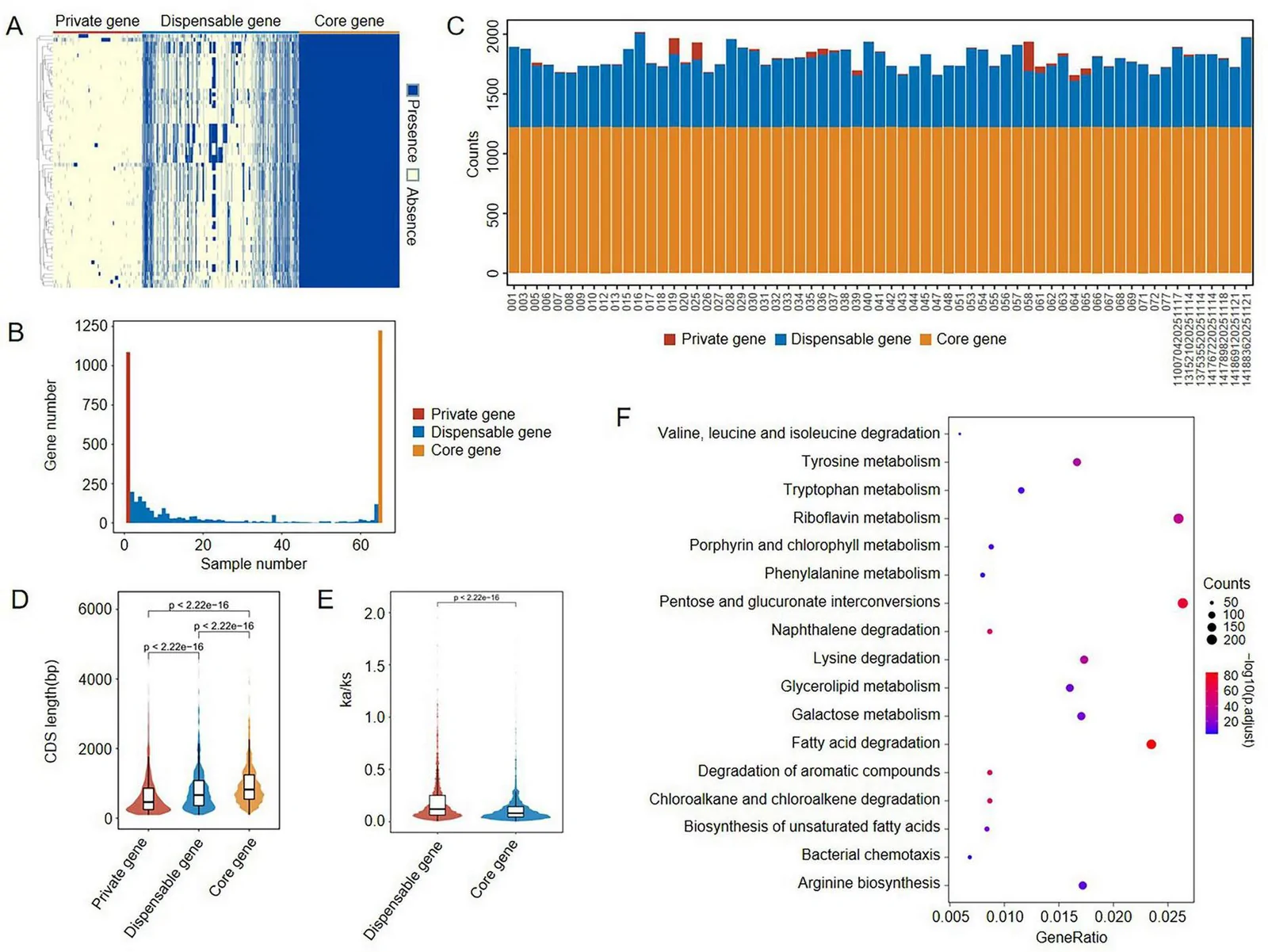
Pangenome structure and functional features of variable gene content in respiratory *H. influenzae*. **(A)** Heatmap showing the presence/absence patterns of Roary-defined pangenome gene clusters across the 65 *H. influenzae* isolates. Each column represents a gene cluster and each row represents an isolate. Gene clusters are categorized as core, dispensable, or private according to their distribution across the isolate collection. Isolates were hierarchically clustered according to their gene cluster presence/absence profiles, allowing patterns of shared and variable gene content among isolates to be visualized. **(B)** The bar chart displays the number of Roary-defined pangenome gene clusters belonging to the core, dispensable, and private gene categories. the x-axis represents the number of isolates, and the y-axis represents the number of genes present in a specific number of isolates. **(C)** The stacked bar chart displays the distribution of core, dispensable, and private gene clusters across the 65 isolates. The x-axis represents each isolate, while the y-axis represents the number of core, dispensable, and private gene clusters. **(D)** The violin plot displays the distribution of coding sequence (CDS) lengths for core, dispensable, and private gene clusters. Pairwise comparisons between the three gene-cluster categories were performed using the Wilcoxon rank-sum test (wilcox.test). *P* values for the pairwise comparisons are shown in the Figure. The comparison illustrates differences in sequence-length characteristics among conserved and variable components of the pangenome. **(E)** The violin plot displays the distribution of Ka/Ks ratios for core and dispensable gene clusters. Statistical significance was assessed using the Wilcoxon rank-sum test. *P* values for the pairwise comparisons are shown in the Figure. Ka/Ks values were calculated to assess the relative patterns of nonsynonymous and synonymous substitutions, with the distribution providing an indication of the evolutionary constraints acting on conserved versus variable gene content. **(F)** The bubble plot displays the results of KEGG pathway enrichment analysis for dispensable gene clusters. Bubble size represents the number of enriched genes, while color indicates the significance of the enrichment results, expressed as -log10 (p.adjust).

Sequence features of different gene cluster categories also reflected functional differentiation between the core and dispensable genomes. CDSs in core gene clusters were significantly longer than those in dispensable and private gene clusters, consistent with their role in conserved fundamental functions (Figure 3D). In contrast, Ka/Ks values were significantly higher for dispensable gene clusters than for core gene clusters (Figure 3E). This pattern indicates greater sequence divergence in the dispensable genome and may reflect stronger adaptive pressure, heterogeneous gene origins, or mobile-element-associated evolution. It should not be interpreted as evidence that all dispensable gene clusters are under positive selection. Functional enrichment of dispensable gene clusters showed that this variation was not randomly distributed across biological functions. GO enrichment identified deoxyribonucleotide metabolism and histidine biosynthesis among enriched biological processes, tRNA methyltransferase activity and diverse transmembrane transporter activities among enriched molecular functions, and extrinsic components of the plasma membrane and translation initiation complex among enriched cellular components (Supplementary Figure 3). These results suggest that the dispensable genome includes not only metabolic genes but also functional modules related to membrane-associated processes and transport. KEGG enrichment further pointed to metabolic flexibility and environmental adaptation. dispensable gene clusters were enriched in xenobiotic degradation, amino acid metabolism, lipid metabolism, bacterial chemotaxis, porphyrin and riboflavin cofactor metabolism, and multiple carbohydrate metabolism pathways (Figure 3F). These functions are consistent with nutrient acquisition, membrane remodeling, environmental response, and transport in the respiratory niche. Compared with the conserved biological processes represented by the core genome (Supplementary Tables 6, 7), the dispensable genome is more likely to contribute to strain-specific adaptation to local microenvironments and antimicrobial pressure.

### Ampicillin resistance is associated with a co-occurring dispensable-genome module

Dispensable-genome differences may influence bacterial adaptation to antimicrobial pressure, but not every gene gain or loss directly explains resistance. To identify dispensable-genome signals associated with β-lactam resistance, we compared the presence or absence of each Roary-defined pangenome gene cluster with four β-lactam phenotypes that showed resistance-category variation. This analysis was based on contingency relationships between gene cluster presence/absence and resistance classification, and evaluated co-occurrence strength using sensitivity, specificity, odds ratio, and Bonferroni-adjusted significance. Among the four drug phenotypes, significant association signals were concentrated in ampicillin resistance. The uncorrected association screen identified 714 Roary-defined pangenome gene clusters showing nominal associations, of which 58 remained significant after Bonferroni correction. These gene clusters did not produce a similarly broad signal for ampicillin-sulbactam, amoxicillin-clavulanate, or cefuroxime, indicating that ampicillin resistance was more strongly associated with dispensable gene content than were inhibitor-containing combinations or cefuroxime resistance (Table 2; Supplementary Table 8). Several gene clusters were almost exclusively present in ampicillin-resistant isolates. The combination of *bla*, *tnpA*, and *tnpR* was especially informative, with approximately 80% sensitivity, 100% specificity, and infinite odds ratios (Table 2). *bla* encodes a TEM-type β-lactamase and directly corresponds to enzymatic ampicillin resistance. *tnpA* and *tnpR* encode a transposase and resolvase, respectively, suggesting that the genetic background carrying this resistance signal had mobile-element features. Many gene clusters annotated as hypothetical proteins shared the same distribution pattern as *bla*, *tnpA*, and *tnpR*. These included group_1715 to group_1717, group_2170 to group_2177, and group_2714 to group_2749. They appeared together in ampicillin-resistant isolates, were largely absent from susceptible isolates, and formed a consistent presence/absence pattern together with the annotated β-lactamase and transposition-related genes (Figure 4). This co-occurrence suggests that the association screen did not capture multiple independent candidate genes, but rather a variable-gene-content module associated with ampicillin resistance. Some gene clusters showed the opposite direction of association. *xylG* and *xylF* had odds ratios of 0, indicating that they were more frequently present in susceptible isolates and absent from ampicillin-resistant isolates (Table 2). This pattern does not imply that *xylG* or *xylF* directly confer susceptibility. Instead, it suggests that the dispensable-genome background associated with ampicillin resistance may involve replacement, loss, or lineage-associated absence of some metabolism- or transport-related genes. In contrast, *gspA* showed high sensitivity in resistant isolates, reaching 94.1%, but had lower specificity than *bla*, *tnpA*, and *tnpR*. This pattern suggests that *gspA* may belong to a broader resistance-associated genetic background rather than acting as a core determinant of ampicillin resistance (Table 2). Ampicillin resistance therefore showed a dispensable genome pattern distinct from that of cefuroxime resistance. Cefuroxime resistance was mainly explained by PBP3 target variation, whereas ampicillin resistance was associated not only with *blaTEM* but also with a set of highly co-occurring dispensable gene clusters. This module contained a TEM-type β-lactamase gene, transposition-related genes, and multiple gene clusters annotated as hypothetical proteins, and it formed a consistent presence/absence pattern in resistant isolates (Figure 4). This finding extends the interpretation of ampicillin resistance from a single β-lactamase gene to a dispensable genome module and suggests that the module may reside in a mobile genetic region associated with resistance distribution.

**TABLE 2:** Roary-defined pangenome gene clusters associated with ampicillin resistance based on gene cluster presence/absence–phenotype association analysis.

Gene	Sensitivity	Specificity	Odds_ratio	Annotation	Bonferroni_p
lrp_2	1.960784	27.27273	0.0075	Leucine-responsive regulatory protein	0.00125
tnpA	80.39216	100	Inf	Tn3 family transposase Tn2	0.002078
bla	80.39216	100	Inf	Beta-lactamase TEM	0.002078
tnpR_2	80.39216	100	Inf	Transposon Tn3 resolvase	0.002078
xylG	21.56863	0	0	Xylose import ATP-binding protein XylG	0.004155
group_2176	76.47059	100	Inf	Hypothetical protein	0.007964
group_1908	74.5098	100	Inf	Hypothetical protein	0.014704
group_1338	74.5098	100	Inf	Hypothetical protein	0.014704
group_1715	74.5098	100	Inf	Hypothetical protein	0.014704
group_1716	74.5098	100	Inf	Hypothetical protein	0.014704
group_1717	74.5098	100	Inf	Hypothetical protein	0.014704
group_2170	74.5098	100	Inf	Hypothetical protein	0.014704
group_2171	74.5098	100	Inf	Hypothetical protein	0.014704
group_2172	74.5098	100	Inf	Hypothetical protein	0.014704
group_2173	74.5098	100	Inf	Hypothetical protein	0.014704
group_2174	74.5098	100	Inf	Hypothetical protein	0.014704
group_2175	74.5098	100	Inf	Hypothetical protein	0.014704
group_2177	74.5098	100	Inf	Hypothetical protein	0.014704
ssb_2	74.5098	100	Inf	Single-stranded DNA-binding protein	0.014704
group_2714	74.5098	100	Inf	Hypothetical protein	0.014704
group_2715	74.5098	100	Inf	Hypothetical protein	0.014704
group_2716	74.5098	100	Inf	Hypothetical protein	0.014704
group_2717	74.5098	100	Inf	Hypothetical protein	0.014704
group_2718	74.5098	100	Inf	Hypothetical protein	0.014704
group_2719	74.5098	100	Inf	Hypothetical protein	0.014704
group_2720	74.5098	100	Inf	Hypothetical protein	0.014704
group_2721	74.5098	100	Inf	Hypothetical protein	0.014704
group_2725	74.5098	100	Inf	Hypothetical protein	0.014704
group_2726	74.5098	100	Inf	Hypothetical protein	0.014704
group_2727	74.5098	100	Inf	Hypothetical protein	0.014704
group_2728	74.5098	100	Inf	Hypothetical protein	0.014704
group_2729	74.5098	100	Inf	Hypothetical protein	0.014704
group_2730	74.5098	100	Inf	Hypothetical protein	0.014704
group_2731	74.5098	100	Inf	Hypothetical protein	0.014704
group_2732	74.5098	100	Inf	Hypothetical protein	0.014704
group_2733	74.5098	100	Inf	Hypothetical protein	0.014704
group_2734	74.5098	100	Inf	Hypothetical protein	0.014704
group_2735	74.5098	100	Inf	Hypothetical protein	0.014704
group_2736	74.5098	100	Inf	Hypothetical protein	0.014704
group_2737	74.5098	100	Inf	Hypothetical protein	0.014704
group_2738	74.5098	100	Inf	Hypothetical protein	0.014704
group_2739	74.5098	100	Inf	Hypothetical protein	0.014704
group_2740	74.5098	100	Inf	Hypothetical protein	0.014704
topB_2	74.5098	100	Inf	DNA topoisomerase 3	0.014704
group_2743	74.5098	100	Inf	Hypothetical protein	0.014704
group_2744	74.5098	100	Inf	Hypothetical protein	0.014704
group_2745	74.5098	100	Inf	Hypothetical protein	0.014704
group_2746	74.5098	100	Inf	Hypothetical protein	0.014704
group_2747	74.5098	100	Inf	Hypothetical protein	0.014704
dnaB_1	74.5098	100	Inf	Replicative DNA helicase DnaB	0.014704
group_2749	74.5098	100	Inf	Hypothetical protein	0.014704
tnpR_1	74.5098	100	Inf	Transposon gamma-delta resolvase	0.014704
group_729	74.5098	100	Inf	Hypothetical protein	0.014704
xylF	25.4902	0	0	D-xylose-binding periplasmic protein	0.014704
gspA	94.11765	72.72727	42.66667	General stress protein A	0.020657
group_1100	100	54.54545	Inf	Hypothetical protein	0.022501
group_220	100	54.54545	Inf	Hypothetical protein	0.022501
group_2722	72.54902	100	Inf	Hypothetical protein	0.026257

Each Roary-defined pangenome gene cluster was tested for association between its presence/absence pattern and antimicrobial resistance phenotype. The table lists gene cluster names, sensitivity, specificity, odds ratios, functional annotations and Bonferroni-adjusted *P* values. Significant associations were detected only for ampicillin resistance. The associated gene clusters included *bla*, *tnpA*, *tnpR*, *xylG*, *xylF*, *gspA* and multiple gene clusters annotated as hypothetical proteins. The co-occurrence of *bla*, transposition-related genes and multiple hypothetical protein gene clusters suggests that the ampicillin resistance-associated signal corresponds to a shared variable-genome module.

**FIGURE 4:**
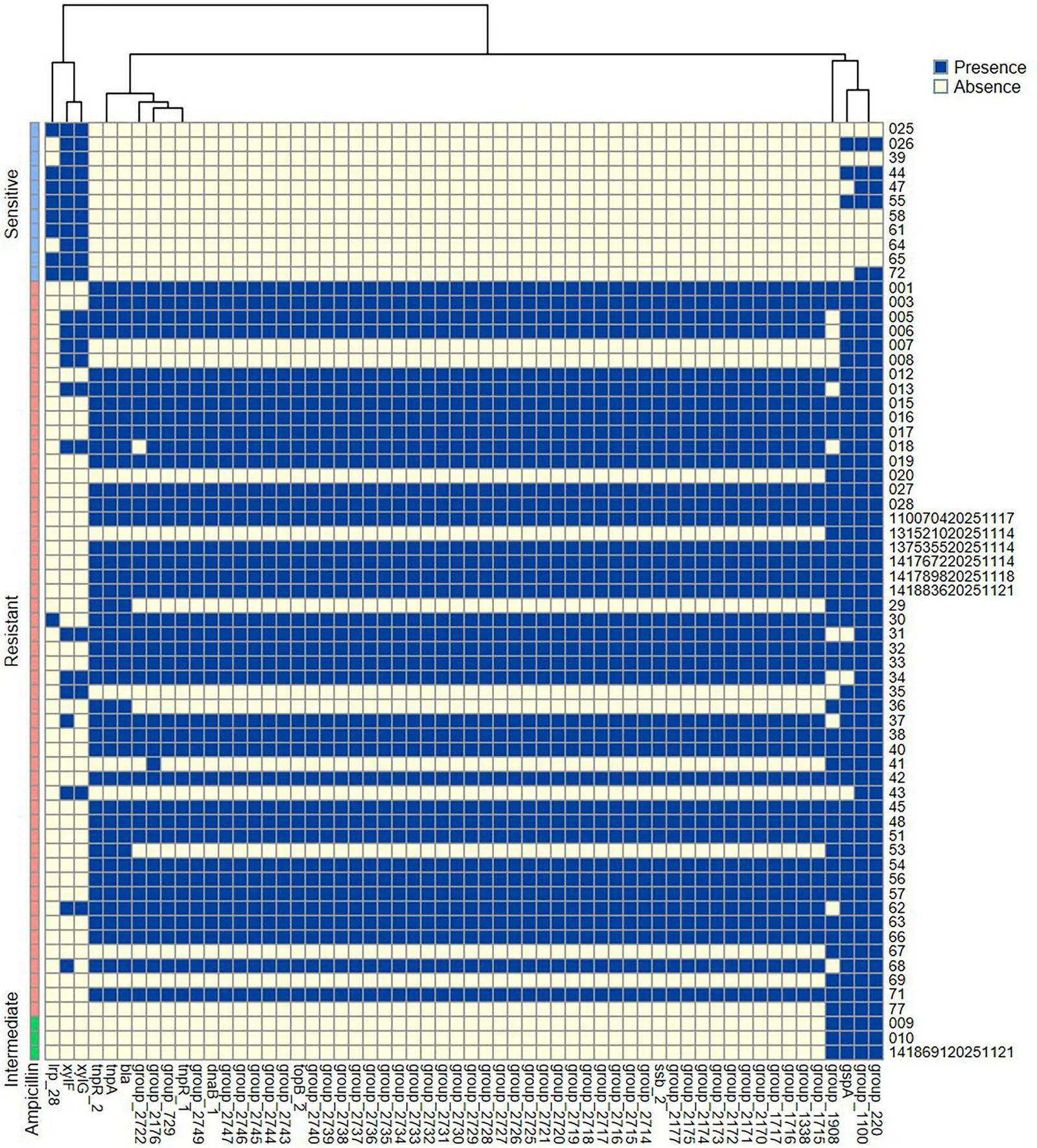
Ampicillin resistance-associated Roary-defined pangenome gene clusters identified by gene cluster presence/absence–phenotype association analysis. Heatmap showing the distribution of Roary-defined pangenome gene clusters significantly associated with ampicillin resistance across the 65 isolates. These gene clusters include *bla*, *tnpA*, *tnpR* and multiple gene clusters annotated as hypothetical proteins. Isolate phenotypes are indicated by color: blue, susceptible; red, resistant; green, intermediate. The co-occurrence pattern of these gene clusters suggests that the ampicillin resistance-associated signal is concentrated in a shared variable-genome module rather than in isolated genes.

### The ampicillin resistance-associated module is embedded in an ICEHinR2866-like mobile genetic region

The coordinated presence of ampicillin resistance-associated gene clusters suggested that these signals might originate from the same dispensable genomic region. To evaluate the structural basis of this inference, we traced the genomic positions of the significant association signals. Fifty gene clusters, including *bla*, were adjacent in genomic coordinates and were usually located on the same contig. This spatial clustering refined the presence/absence association signal from statistical co-occurrence to a continuous genomic region rather than independent variation at multiple dispersed loci. This region showed a conserved organization across different *blaTEM-1*-positive isolates. Synteny comparison of four representative *blaTEM-1*-positive genomes revealed similar gene order and high sequence similarity among resistance-associated genes, although local gene losses were present in individual isolates (Figure 5A). This conserved organization indicates that the ampicillin resistance-associated module likely exists as a genomic segment rather than as independent random insertions of a single resistance gene into different backgrounds. Integrative and Conjugative Element (ICE) annotation further revealed the genetic nature of this region. One side of the region contained integrase and relaxase genes, whereas the other side contained T4SS-associated components, consistent with the typical features of an integrative and conjugative element (Figure 5B). In the middle of the element, *blaTEM-1B* co-occurred with *mef(A)* and an ABC-F type ribosomal protection protein, indicating that the region carried not only a β-lactamase gene but also macrolide resistance-associated determinants. Thus, the ampicillin resistance-associated module extended from a single β-lactamase gene to an ICE-like region containing multiple resistance-related genes and mobile-element components. Comparison with known ICE sequences further clarified the relationship of this element to established ICE families. The region was similar to multiple ICEHin1056 family elements in ICEberg3 and showed the highest similarity to ICEHinR2866. Accordingly, this resistance-associated region can be classified as an ICEHinR2866/ICEHin1056-like element (Figure 5B). This structural feature is consistent with the polyclonal distribution of β-lactamase positivity and supports the potential for the *blaTEM-1B*-associated ampicillin resistance module to occur across different genetic backgrounds through a mobile genetic element. Taken together, the ampicillin resistance-associated signal did not remain a gene presence/absence-level statistical association. It was mapped to a continuous genomic region with mobile-element architecture. This putative ICEHinR2866/ICEHin1056-like region contains *blaTEM-1B*, transposition-associated components, conjugation-associated components, and additional resistance-associated genes, providing a plausible structural basis for the organization of the ampicillin resistance-associated module in this cohort.

**FIGURE 5:**
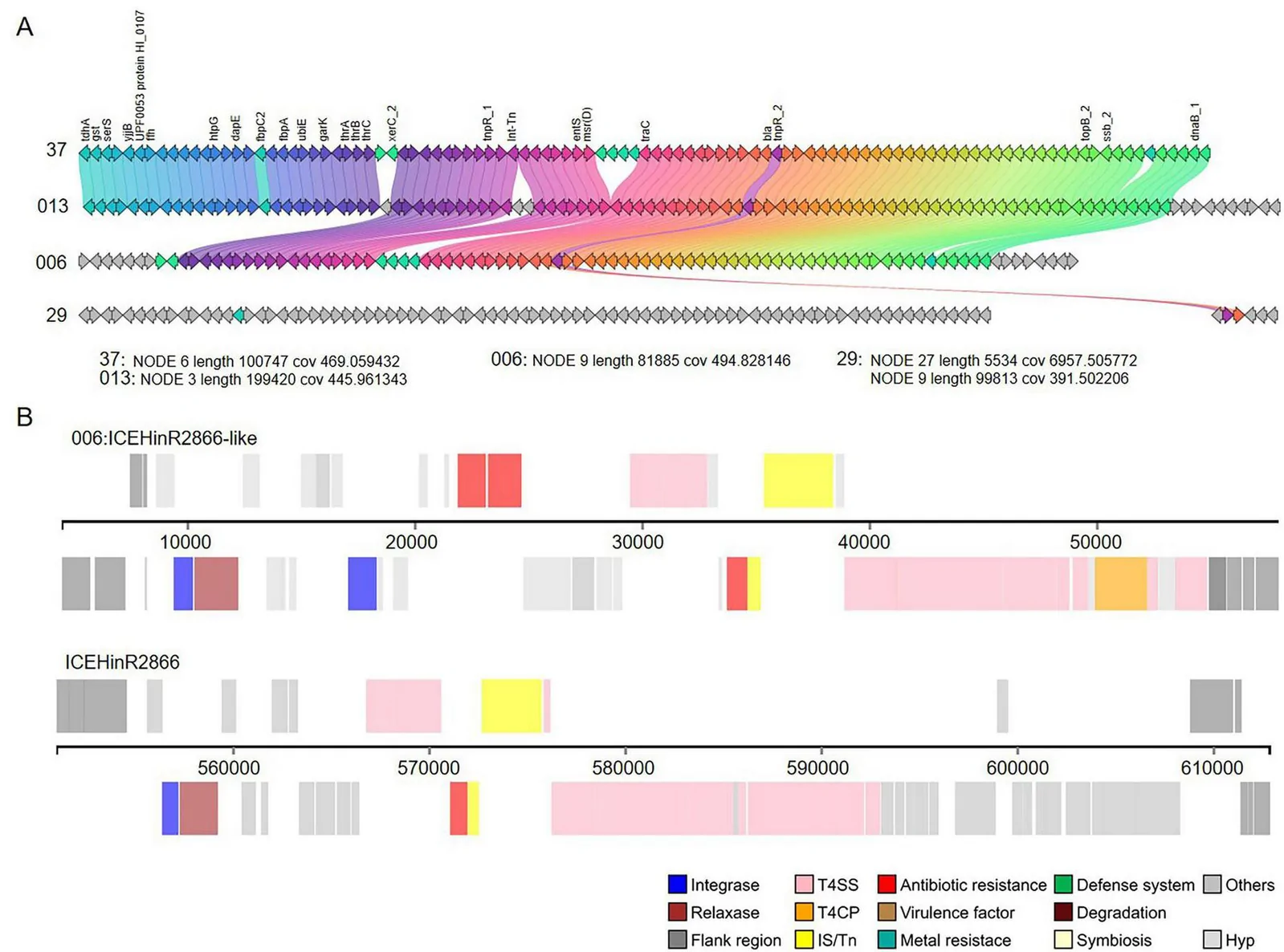
Synteny and structural organization of an ICEHinR2866/ICEHin1056-like resistance-associated region. **(A)** Synteny comparison of ampicillin resistance-associated gene regions in four representative *bla*TEM-1-positive isolates. Homologous genes are connected between isolates to indicate sequence similarity and conserved gene order. A local gene loss is observed in isolate 29. **(B)** Structural organization of the ICE-like region identified in isolate 6 and the known ICE region. The gene map indicates predicted ICE-associated components, including integrase, relaxase and T4SS-associated genes, together with resistance-associated determinants including *bla*TEM-1B, *mef(A)* and an ABC-F type ribosomal protection protein. Sequence comparison with ICEberg3 entries indicates that this region is most similar to ICEHinR2866 and belongs to an ICEHin1056-like family.

## Materials and methods

### Isolation of *H. influenzae*

Between September 2025 and January 2026, researchers isolated *H. influenzae* from sputum and bronchoalveolar lavage fluid of pneumonia patients at Chengdu Fifth People’s Hospital. A total of 65 *H. influenzae* isolates were obtained. Only one *H. influenzae* isolate was collected and included from each patient; therefore, no repeated isolates from the same patient were included in this study. This study was approved by the Ethics Committee of Chengdu Fifth People’s Hospital (Approval No. 2024-047-01). Written informed consent was obtained from all participants. The isolates were identified as *H. influenzae*. Positive colonies were purified by continuous streaking on chocolate agar plates, and pure cultures were obtained for subsequent experiments. Subsequently, the nitrocefin disk method was used to detect whether the colonies produced β-lactamase, in order to evaluate the antimicrobial resistance of the isolates. Finally, the identified isolates were stored at −80 °C.

### DNA extraction and whole-genome sequencing

Genomic DNA was extracted from each *H. influenzae* isolate using the Solarbio bacterial genomic DNA extraction kit. DNA quality and quantity were evaluated using Nanodrop spectrophotometry, Qubit fluorometric quantification, and agarose gel electrophoresis. Qualified genomic DNA was randomly fragmented to the target insert size using fragmentation enzyme treatment. The fragmented DNA was subjected to end repair, dA-tailing, adapter ligation, magnetic bead-based purification, and PCR enrichment. The amplified libraries were re-evaluated for concentration and fragment-size distribution. After denaturation, circularization was performed, and non-circularized linear DNA was digested enzymatically. Libraries were pooled according to the target sequencing depth and sequenced on the MGI DNBSEQ platform to generate paired-end reads.

### Antimicrobial susceptibility testing

Antimicrobial susceptibility was determined by the minimum inhibitory concentration (MIC) method. The following 12 antimicrobial agents were tested: Ampicillin, Ampicillin-Sulbactam, Amoxicillin-Clavulanate, Cefuroxime, Ceftriaxone, Cefepime, Cefixime, Aztreonam, Meropenem, Lomefloxacin, Levofloxacin, and Azithromycin. Susceptibility categories were assigned as resistant, intermediate, or susceptible according to the Clinical and Laboratory Standards Institute (CLSI) standard M100, 35th edition (M100-S35, 2025), Performance Standards for Antimicrobial Susceptibility Testing. For genotype–phenotype concordance analyses, isolates classified as intermediate were excluded from the binary resistant-versus-susceptible comparison. Therefore, the sample size used for Cohen’s Kappa analysis could vary among antimicrobial agents depending on the distribution of susceptibility categories. Detailed susceptibility classifications for each isolate are provided in Supplementary Table 1.

### Genome assembly and quality assessment

Raw sequencing reads were filtered using fastp v0.23.4 (Chen et al., 2018) to remove adapters, low-quality bases, and short reads. PCR duplicates in paired-end reads were removed using FastUniq (Xu et al., 2012). De novo genome assembly was performed with SPAdes v4.2.0 using the – isolate option (Vasilinetc et al., 2015). The assembled scaffolds were classified using Kraken2 with the pluspf16g database, and target-species reads or scaffolds were extracted using extract_kraken_reads.py to remove potential non-*H. influenzae* contamination.

For gap closing, clean reads were mapped back to the corresponding draft genome using Burrows-Wheeler Aligner BWA v0.7.17-r1188 (Li and Durbin, 2009). SAMtools v1.13 stats was used to estimate the average insert size from the resulting BAM files (Danecek et al., 2021), and SOAPdenovo2 (Luo et al., 2012) GapCloser v1.12 was then run with the estimated insert-size information to iteratively close assembly gaps. Genome assembly quality was evaluated using QUAST v5.2.0 to calculate assembly metrics including N50, L50, genome size, GC content, and maximum contig length (Gurevich et al., 2013). Completeness was assessed using BUSCO v6.0.0 with the bacteria_odb12 database (Tegenfeldt et al., 2025).

### Gene prediction and functional annotation

Protein-coding genes, tRNAs, rRNAs, and tmRNAs were annotated using Prokka v1.13 (Seemann, 2014). Functional annotation of predicted protein-coding genes was performed using EggNOG-mapper v2.1.2 (Cantalapiedra et al., 2021), from which Gene Ontology (GO) annotations were extracted. Kyoto Encyclopedia of Genes and Genomes (KEGG) pathway annotation was performed using KOBAS v2.0. Pfam domains were identified using HMMER hmmsearch v3.4 (Eddy, 2023).

### MLST typing and core-genome phylogenetic analysCCis

Multilocus sequence typing was performed using MLST v2.33.1 against the PubMLST database (Jolley et al., 2018). Allelic profiles and sequence types were assigned according to the *H. influenzae* MLST scheme deposited in PubMLST. The resulting ST profiles were uploaded to PHYLOViZ to construct a minimum spanning tree for evaluating genetic relatedness among STs.

To resolve genome-wide phylogenetic relationships among isolates, the H. influenzae reference genome GCF_020736045.1 was downloaded from NCBI and used as the reference for core-genome single nucleotide polymorphism analysis. Core-genome SNPs were identified using Snippy. Recombinant regions were removed from the alignment using Gubbins v3.0.0 (Croucher et al., 2015). A maximum-likelihood phylogenetic tree was constructed using FastTree v2.2.0 with default parameters and visualized using iTOL (Price et al., 2010).

### Antimicrobial resistance gene detection and genotype-phenotype concordance analysis

Antimicrobial resistance genes and resistance-associated point mutations were identified using AMRFinderPlus v4.0.15 with the NCBI AMR gene database version 2024-12-18.1 (Feldgarden et al., 2021). Resistance genes were called using the default AMRFinderPlus thresholds, with minimum sequence identity of 90% and minimum coverage of 80%.

To evaluate the concordance between genotypic markers and antimicrobial resistance phenotypes, each detected resistance gene or mutation was compared with the resistance phenotype of each antimicrobial agent. Cohen’s Kappa coefficient was calculated using the R package irr. Fisher’s exact test and odds ratio were used to evaluate the strength of association between each genotype and resistance phenotype. Genotype–phenotype pairs with Kappa ≥ 0.4 were considered to show moderate or stronger concordance and were further examined. Because only a subset of tested antimicrobial agents showed informative resistant and susceptible categories in this isolate collection, the main concordance analysis focused on Ampicillin, Ampicillin-Sulbactam, Amoxicillin-Clavulanate, and Cefuroxime.

### Pangenome construction and evolutionary analysis of gene categories

GFF files generated by Prokka were used as input for Roary pangenome analysis. Roary was run with a minimum BLASTP identity threshold of 80% (Page et al., 2015). Based on gene presence across the 65 isolates, pangenome gene clusters were classified into core genes, dispensable genes, and private genes. Core genes were defined as gene clusters present in all 65 isolates. Dispensable genes were defined as gene clusters present in 2–64 isolates. Private genes were defined as gene clusters present in only one isolate.

CDS and protein sequences of each gene category were extracted from the Roary clustering results. Pairwise nonsynonymous substitution rate (Ka) and synonymous substitution rate (Ks) were calculated using KaKs_Calculator v2.1.0 (Wang et al., 2010). Functional enrichment analysis of core and dispensable genes was performed using clusterProfiler based on GO and KEGG annotations.

### Gene presence/absence-phenotype association analysis

To identify dispensable gene clusters associated with antimicrobial resistance phenotypes, the pangenome gene presence/absence matrix was compared with resistance phenotypes for antibiotics that showed informative variation among isolates. This analysis was performed using Scoary v1.6.16 (Brynildsrud et al., 2016). For each gene cluster, its presence or absence was compared with the binary resistance phenotype, and sensitivity, specificity, odds ratio, and *P* value were calculated. Multiple testing correction was performed using the Bonferroni method, and gene clusters with Bonferroni-adjusted *P* ≤ 0.05 were considered significantly associated with the corresponding resistance phenotype.

### Identification and comparative analysis of integrative and conjugative elements

Integrative and conjugative elements were predicted using locally deployed ICEfinder2 v2.0 across the assembled contigs of the 65 isolates (Wang et al., 2024). Based on the gene presence/absence–phenotype association results, genomic regions containing target resistance-associated gene clusters were extracted in GenBank format. Gene cluster organization and structural conservation among representative isolates were compared using clinker v0.0.32 (Gilchrist and Chooi, 2021).

To assess the relationship between the identified ICE-like regions and previously reported ICEs, known ICE sequences were downloaded from the ICEberg3 database. BLASTN was used to compare the identified ICE-like sequences with ICEberg3 reference sequences, and sequence similarity was used to infer their relationship to known ICE families.

## Discussion

The use of Hib conjugate vaccines has substantially reshaped the disease ecology of *H. influenzae*. As the burden of classical Hib invasive disease has declined, the clinical importance of *H. influenzae* in respiratory infections and some invasive disease settings has increased, with antimicrobial resistance being particularly prominent. Reviews have emphasized that *H. influenzae* is not only a major cause of otitis media, sinusitis, and acute exacerbations of chronic obstructive pulmonary disease, but can also cause bacteremia, meningitis, and pneumonia in older adults, immunocompromised individuals, and other high-risk hosts, and the evolving antimicrobial resistance has further complicated the treatment of these infections. Thus, research on *H. influenzae* in the post-Hib vaccine era needs to move beyond single-serotype invasiveness and address how genetically diverse respiratory populations persist in mucosal niches, acquire resistance, and generate clinically relevant risk (Langereis and de Jonge, 2015). This study focused on that problem. *blaTEM*-mediated β-lactamase production and *ftsI*/PBP3-mediated target alteration are established mechanisms of β-lactam resistance in *H. influenzae*. However, knowing these mechanisms does not fully explain how they are distributed and genetically organized within clinical populations. The same resistance phenotype can arise through clonal expansion or repeated occurrence of resistance determinants across multiple genetic backgrounds. Resistance genes can also exist as isolated markers or be embedded in dispensable genome regions and ICE-like mobile elements. By analyzing susceptibility phenotypes in the context of phylogeny, pangenome structure, and putative mobile genetic elements, this study extends the interpretation of β-lactam resistance from individual determinants to population-scale genetic organization.

Our findings are consistent with previous work showing high genetic diversity in *H. influenzae*. Large-scale genomic studies have shown that *H. influenzae* has complex local and global population structures, and that resistant lineages can occur across different genetic backgrounds (Frank et al., 2024; Zhang et al., 2024). The 65 respiratory isolates analyzed here similarly formed a polyclonal population, with 28 known STs and 12 unassigned STs, and the core-genome phylogeny showed multiple diverging branches. Importantly, β-lactamase-positive isolates did not form an independent phylogenetic group but were interspersed with β-lactamase-negative isolates across different branches. This pattern does not support single-clone expansion as the main explanation for β-lactamase positivity. It is more consistent with the acquisition or maintenance of resistance determinants across multiple genetic backgrounds. When a β-lactamase-positive phenotype crosses multiple STs and core-genome branches, the genetic vehicle and background of the resistance determinant become central to the interpretation of resistance distribution. Previous studies have shown that *H. influenzae* can acquire resistance through chromosomal mutation, recombination, plasmids, transposable elements, and ICEs. ICEHin1056 and related elements are especially recognized as platforms for antibiotic resistance gene distribution in *Haemophilus* (Mohd-Zain et al., 2004; Juhas et al., 2013). The polyclonal distribution of β-lactamase positivity in this study therefore provided the population-genetic context for tracing resistance modules in the dispensable genome and mobile elements.

At the mechanistic level, our results agree with the established dual basis of β-lactam resistance in *H. influenzae*. β-lactamase genes such as *blaTEM-1* and *blaROB-1* can mediate ampicillin and amoxicillin resistance, whereas amino acid substitutions in PBP3 encoded by *ftsI* reduce target affinity for β-lactams and can produce β-lactamase-negative ampicillin resistance or reduced cephalosporin susceptibility (Tristram et al., 2007; Diricks et al., 2024). In this cohort, *blaTEM-1* showed strong concordance with ampicillin resistance, whereas PBP3 substitutions S357N, S385T, and L389F were significantly associated with cefuroxime resistance. These results indicate that ampicillin resistance in this cohort was mainly influenced by enzymatic resistance, whereas cefuroxime resistance more clearly reflected PBP3 target alteration.

A more important conclusion is that these known markers were strongly associated with resistance but were not fully predictive. *blaTEM-1* explained most ampicillin resistance but was not detected in all resistant isolates. The lower concordance between *blaTEM-1* and ampicillin-sulbactam resistance further shows that β-lactamase inhibitors alter the relationship between a single gene and a susceptibility phenotype. PBP3 substitutions showed a similar pattern. Cefuroxime-resistant isolates all carried key *ftsI*-encoded PBP3 substitutions, but some isolates carrying these substitutions remained susceptible. Previous work has also shown that β-lactamases can confer high-level resistance to penicillins and early cephalosporins, whereas PBP3 substitutions often produce low- to intermediate-level cross-resistance, and that existing PBP3 groupings do not fully explain all phenotypic differences (Diricks et al., 2024). Such incomplete correspondence indicates that molecular resistance prediction should not rely on single genes or isolated substitutions alone, but should incorporate composite mutation profiles, drug combinations, genetic background, and additional mechanisms.

The *blaTEM*-negative but ampicillin-resistant isolates illustrate this complexity. These atypical isolates carried similar composite *ftsI* mutation profiles encoding PBP3 substitutions S357N, S385T, M377I, R517H, and D350N, together with additional substitutions such as L389F and Y557H. Clinical studies have also reported PBP3 alterations in β-lactamase-negative ampicillin-resistant isolates, β-lactamase-positive amoxicillin-clavulanate-resistant isolates, and other β-lactamase-positive resistant strains (Frank et al., 2024). The composite *ftsI* profiles observed in this cohort should therefore not be interpreted as an alternative to the *blaTEM* mechanism, but as another genetic layer that can act together with enzymatic resistance within the β-lactam resistance spectrum.

Pangenome analysis further showed that these resistance phenotypes exist within a highly plastic dispensable genome. The isolates shared a relatively stable core genome, but dispensable and private gene clusters were abundant. dispensable gene clusters were enriched in functions related to transmembrane transport, membrane-associated components, metabolism, bacterial chemotaxis, and cofactor metabolism. These observations do not prove that any individual variable gene confers a resistance advantage. They do show, however, that resistance in respiratory *H. influenzae* occurs within a genetically heterogeneous population rather than a uniform genomic background. Instead, it is embedded in a dispensable genome space shaped by nutrient acquisition, membrane remodeling, transport, and local adaptation. Private gene clusters represented an additional and more extreme layer of genomic variability in this population. In contrast to dispensable genes, which were shared by multiple isolates, private genes were detected in only a single isolate and therefore represented highly strain-specific gene content. Their presence may reflect recent gene acquisition, lineage-specific genomic diversification, or the accumulation of genetic material associated with mobile elements and other localized genomic events. The concentration of private gene clusters in several isolates (particularly isolates 19, 25, and 58) suggests that some strains may harbor distinctive accessory-genome components that are not broadly distributed within the sampled population. However, because private genes occur in only one isolate by definition, their biological functions and potential contributions to host adaptation or antimicrobial resistance cannot be reliably inferred from the current dataset. Further studies involving larger collections of H. influenzae isolates, functional annotation, comparative genomics, and experimental validation will be required to determine whether individual private genes contribute to niche adaptation, persistence, or other clinically relevant phenotypes.

The gene cluster presence/absence–phenotype association screen focused this dispensable-genome background on ampicillin resistance. This analysis should be interpreted carefully. It is not equivalent to SNP-GWAS and cannot by itself prove causality. Rather, it measures the co-occurrence between each Roary-defined pangenome gene cluster and a resistance classification. In this sense, the concentration of 58 significant gene clusters in ampicillin resistance, with no comparable signal for ampicillin-sulbactam, amoxicillin-clavulanate, or cefuroxime, suggests that ampicillin resistance was highly co-occurring with a specific dispensable-genome module. The most informative components of this module were *bla*, *tnpA*, and *tnpR*. *bla* corresponds to a TEM-type β-lactamase, whereas *tnpA* and *tnpR* encode transposition-related functions. Many gene clusters annotated as hypothetical proteins shared the same presence/absence pattern. Thus, the association screen did not identify a set of independent candidate resistance genes, but pointed to a continuous or co-transmitted genomic region. This shifts the interpretation of ampicillin resistance from detection of *blaTEM* alone to the organization of the variable genetic background carrying *blaTEM*.

ICE analysis anchored this interpretation to a specific genetic vehicle. Multiple ampicillin resistance-associated gene clusters, including *bla*, were adjacent in genomic coordinates and showed conserved synteny in multiple *blaTEM-1*-positive isolates. The region carried integrase, relaxase, and T4SS-associated components and was most similar to ICEHinR2866 within the ICEHin1056 family. Previous studies have shown that ICEHin1056-related elements are modular and can participate in horizontal transfer of antibiotic resistance genes in *H. influenzae* and related *Haemophilus* species (Mohd-Zain et al., 2004; Juhas et al., 2013). The ICEHinR2866/ICEHin1056-like region identified here therefore provides a plausible structural basis for the occurrence of β-lactamase positivity across multiple genetic backgrounds.

This ICE-like region is notable because it does not carry *blaTEM-1B* alone. It also contains *mef(A)* and an ABC-F type ribosomal protection protein associated with macrolide resistance. Similar clustering of multiple resistance genes has been reported in *H. influenzae* ICEs. For example, Tn7100 has been described as an ICE carrying *blaTEM-1B*, *catA2*, *aac(6′)-Im*, *aph(2″)-Ib*, *mef(E)*, and *mel*, with circularization and low-frequency transfer capacity (Dimopoulou et al., 2007; Juhas et al., 2013). The ampicillin resistance module detected in this study may therefore not be maintained solely by aminopenicillin pressure. If β-lactam and macrolide resistance determinants are located in the same mobile genetic region, use of different antimicrobial classes could indirectly select for retention of the same element. This genetic linkage has direct implications for antimicrobial stewardship. Empirical therapy often treats resistance mechanisms for different drug classes separately, but mobile elements can assemble multiple determinants on the same genetic platform. For respiratory *H. influenzae*, which can persistently colonize mucosal sites and experience repeated antimicrobial exposure, the genomic context of a resistance gene can be as important as its presence. Molecular surveillance focused only on the presence of *blaTEM* would not reveal whether it is embedded in an ICE-like region with distribution potential, and β-lactamase testing alone would miss non-enzymatic resistance mediated by composite PBP3 substitutions.

The dissemination of antimicrobial resistance is determined not only by the resistance phenotype and mobility of the underlying determinants, but also by their effects on bacterial fitness. Diricks et al. (2024) proposed that some PBP3 substitutions detected in resistant haplotypes, including M377I, may exert compensatory or other fitness-related effects rather than independently increasing β-lactam resistance. Johannessen et al. (2022) showed that acquisition of the multidrug-resistance ICE Tn7100 reduced the competitive fitness of *H. influenzae*, although isolates carrying this element were still capable of clonal spread. Petersen et al. subsequently demonstrated that most experimentally evolved β-lactam-resistant *H. influenzae* clones had reduced competitive fitness in antibiotic-free conditions, whereas several resistant clones showed no measurable fitness defect, indicating that fitness effects depend on the particular resistance mutations, accompanying mutations and genomic background (Sabine et al., 2025). In our collection, β-lactamase positivity and composite *ftsI*/PBP3 substitution profiles were distributed across multiple phylogenetic backgrounds, suggesting that these determinants are not confined to a single successful resistant lineage. The location of *blaTEM-1B* together with macrolide-resistance determinants in an ICEHinR2866/ICEHin1056-like region also raises the possibility that antibiotic selection and co-selection may offset fitness costs and promote maintenance of the element. Nevertheless, phylogenetic distribution and gene carriage provide only indirect evidence of ecological persistence and cannot quantify fitness. Because our study did not include isogenic strains, growth or competition experiments, the magnitude and direction of the fitness effects associated with individual PBP3 substitutions or the ICE-like region remain unresolved.

Taken together, this study connects previous knowledge of *blaTEM*, PBP3, and ICEs with their organization in a local respiratory isolate population. *blaTEM-1* provided the major enzymatic basis for ampicillin resistance, composite *ftsI*/PBP3 substitution profiles explained cefuroxime and some ampicillin resistance phenotypes, and ampicillin resistance-associated gene clusters were organized within an ICEHinR2866/ICEHin1056-like mobile region. β-lactam resistance in this cohort can therefore be understood as the combined outcome of enzymatic resistance, target remodeling, and a putative mobile genetic platform. This interpretation more closely reflects how resistance forms and is distributed in clinical bacterial populations than a single-marker model.

Several limitations should be considered. The current dataset did not systematically resolve serotypes or capsular loci, so the proportions of NTHi, non-type b encapsulated strains, and Hib among these respiratory isolates cannot be directly determined. Given the biological and vaccine-coverage differences among these groups, future work should include analysis of the cap locus, *bexA-D*, and serotype-specific genes. Another limitation is the use of short-read assemblies. ICE boundaries, integration sites, and complete structures require further validation by long-read closed genomes, breakpoint PCR, or read mapping. Finally, gene presence/absence association screening reveals co-occurrence rather than functional causality. The specific contributions of *blaTEM*, composite *ftsI* mutations, and the ICE-like region to resistance phenotypes require further validation using transformation, allelic replacement, conjugation assays, and standardized MIC testing.

Despite these limitations, this study advances the genomic understanding of β-lactam resistance in respiratory *H. influenzae* from the perspectives of population structure, resistance phenotypes, and mobile genetic elements. It confirms the contribution of classical resistance mechanisms in a local clinical cohort and shows how these mechanisms can be organized as dispensable-genome modules in a polyclonal background. For surveillance of respiratory *H. influenzae* in the post-Hib vaccine era, molecular strategies should move beyond single β-lactamase testing toward integrated analysis of *ftsI*/PBP3 substitution profiles, ICE-like elements, and resistance-associated module structures. Such a WGS-supported framework can improve resistance prediction and help identify resistance platforms with potential to occur across multiple genetic backgrounds.

## Data Availability

The datasets presented in this study can be found in online repositories. The names of the repository/repositories and accession number(s) can be found in the article/Supplementary material.

## Supplementary material

Supplementary Figure 1 The heatmap shows the combined PBP3 substitution profiles in cefuroxime-resistant isolates. Blue indicates susceptible isolates and red indicates resistant isolates.

Supplementary Figure 2 Heatmap showing the presence/absence patterns of Roary-defined pangenome gene clusters across the 65 H. influenzae isolates. Each column represents a gene cluster and each row represents an isolate.

Supplementary Figure 3 GO enrichment results of variable genes. The three panels in the figure display three categories of GO terms: Biological Process (BP), Molecular Function (MF), and Cellular Component (CC).

Supplementary Table 1 Metadata and β-lactamase status of the 65 respiratory *H. influenzae* isolates. This table summarizes isolate identifiers, specimen sources, including sputum and bronchoalveolar lavage fluid, and β-lactamase detection status determined by the nitrocefin disk method.

Supplementary Table 2 Genome assembly statistics of the 65 respiratory *H. influenzae* isolates. This table reports assembly metrics generated by QUAST, including genome size, GC content, N50, L50, maximum contig length and N content.

Supplementary Table 3 BUSCO-based genome completeness assessment. This table summarizes BUSCO completeness statistics for each assembled genome using the bacteria_odb12 database, including complete, single-copy, duplicated, fragmented and missing BUSCO categories.

Supplementary Table 4 The sequence type corresponding to each isolated sample. This table lists the Sequence Type (ST) corresponding to each of the 65 isolates.

Supplementary Table 5 Antimicrobial resistance-associated genes and mutation sites identified by AMRFinderPlus. This table lists antimicrobial resistance genes and resistance-associated point mutations detected in the 65 isolates using AMRFinderPlus v4.0.15 with the NCBI AMR database version 2024-12-18.1.

Supplementary Table 6 GO enrichment results for core genes. This table lists the complete results of the GO enrichment analysis based on 1,224 core genes. Bonferroni-corrected p-values were used to identify results with significant enrichment (p.adjust < 0.05).

Supplementary Table 7 KEGG enrichment results for core genes. This table lists the complete results of the KEGG enrichment analysis based on 1,224 core genes. Bonferroni-corrected p-values were used to identify pathways with significant enrichment (p.adjust < 0.05).

Supplementary Table 8 Gene cluster presence/absence–phenotype association results for antimicrobial resistance traits. This table contains the full association screening results between Roary-defined pangenome gene cluster presence/absence patterns and antimicrobial resistance phenotypes. Bonferroni-adjusted P values were used to identify significantly associated gene clusters.
