# Supplementary material for: Genetic organization and mobile-element basis of β-lactam resistance in a polyclonal respiratory Haemophilus influenzae population
Source: Front Microbiol. 2026 Sep 29;17:1945657. doi: 10.3389/fmicb.2026.1945657 (PMC13660433; doi:10.3389/fmicb.2026.1945657)
Supplement: Supplementary file 1 [file Data_Sheet_1.pdf]

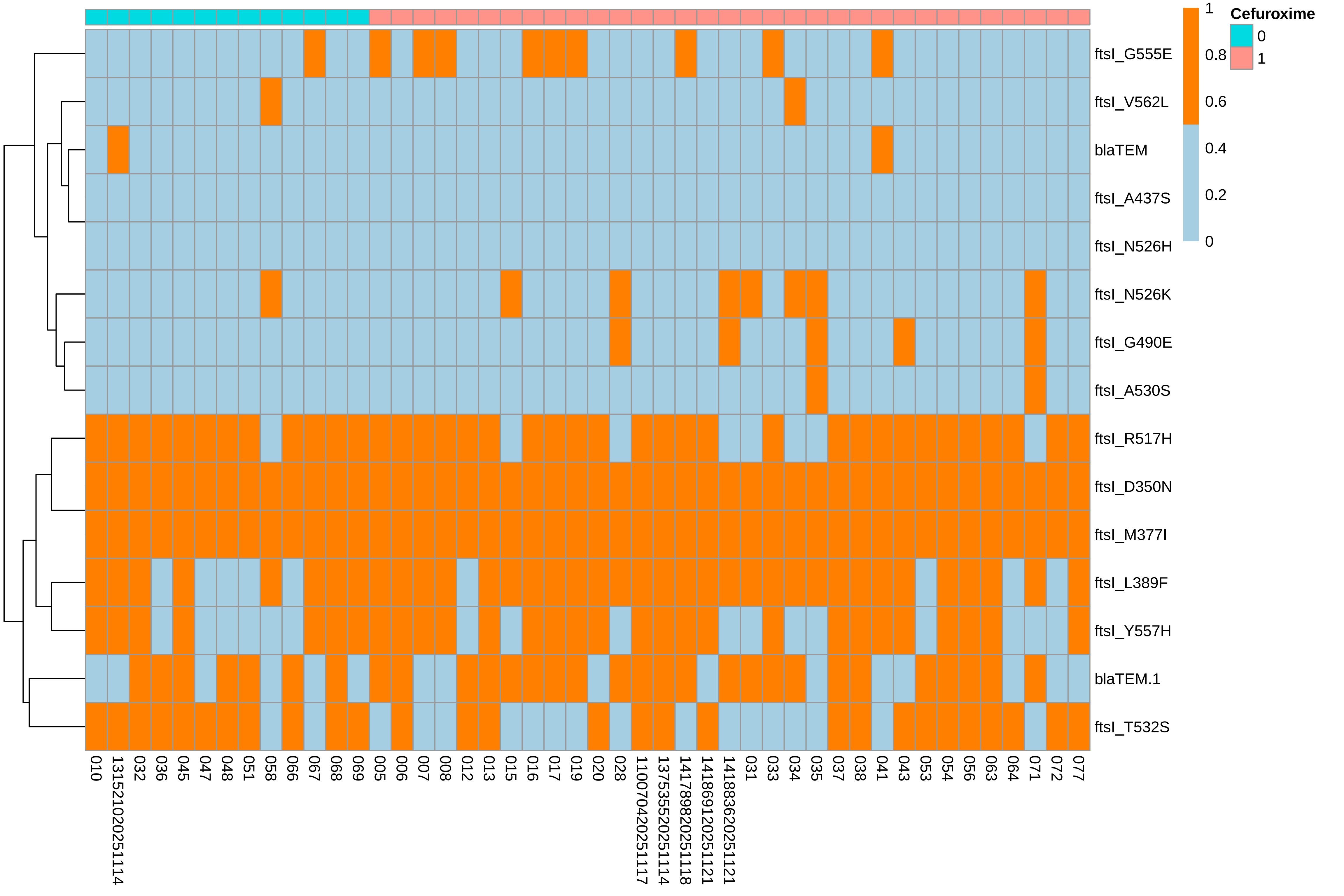
Cefuroxime
0
1
1
0.8
0.6
0.4
0.2
0
ftsI_G555E
ftsI_V562L
blaTEM
ftsI_A437S
ftsI_N526H
ftsI_N526K
ftsI_G490E
ftsI_A530S
ftsI_R517H
ftsI_D350N
ftsI_M377I
ftsI_L389F
ftsI_Y557H
blaTEM.1
ftsI_T532S
010
13152102025111 4
032
036
045
047
048
051
058
066
067
068
069
005
006
007
008
012
013
015
016
017
019
020
028
110070420251117
137535520251114
141789820251118
141869120251121
141883620251121
031
033
034
035
037
038
041
043
053
054
056
063
064
071
072
077
