# Supplementary material for: Genetic organization and mobile-element basis of β-lactam resistance in a polyclonal respiratory Haemophilus influenzae population
Source: Front Microbiol. 2026 Sep 29;17:1945657. doi: 10.3389/fmicb.2026.1945657 (PMC13660433; doi:10.3389/fmicb.2026.1945657)
Supplement: Supplementary file 2 [file Data_Sheet_2.pdf]

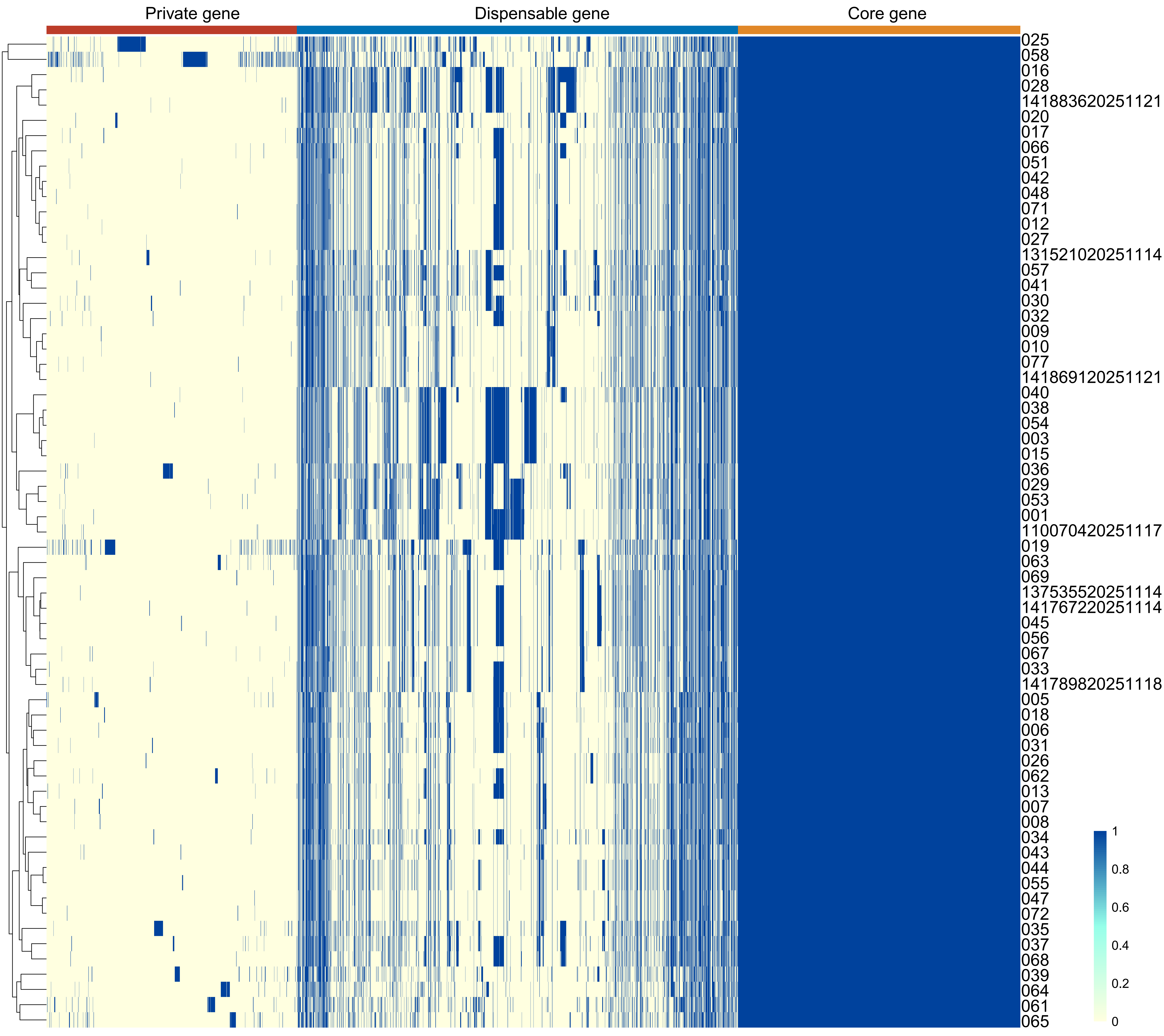

Private gene
Dispensable gene
Core gene
025
058
016
028
141883620251121
020
017
066
051
042
048
071
012
027
131521020251114
057
041
030
032
009
010
077
141869120251121
040
038
054
003
015
036
029
053
001
110070420251117
019
063
069
137535520251114
141767220251114
045
056
067
033
141789820251118
005
018
006
031
026
062
013
007
008
034
043
044
055
047
072
035
037
068
039
064
061
065
1
0.8
0.6
0.4
0.2
0
