# Supplementary material for: Genetic organization and mobile-element basis of β-lactam resistance in a polyclonal respiratory Haemophilus influenzae population
Source: Front Microbiol. 2026 Sep 29;17:1945657. doi: 10.3389/fmicb.2026.1945657 (PMC13660433; doi:10.3389/fmicb.2026.1945657)
Supplement: Supplementary file 3 [file Data_Sheet_3.pdf]

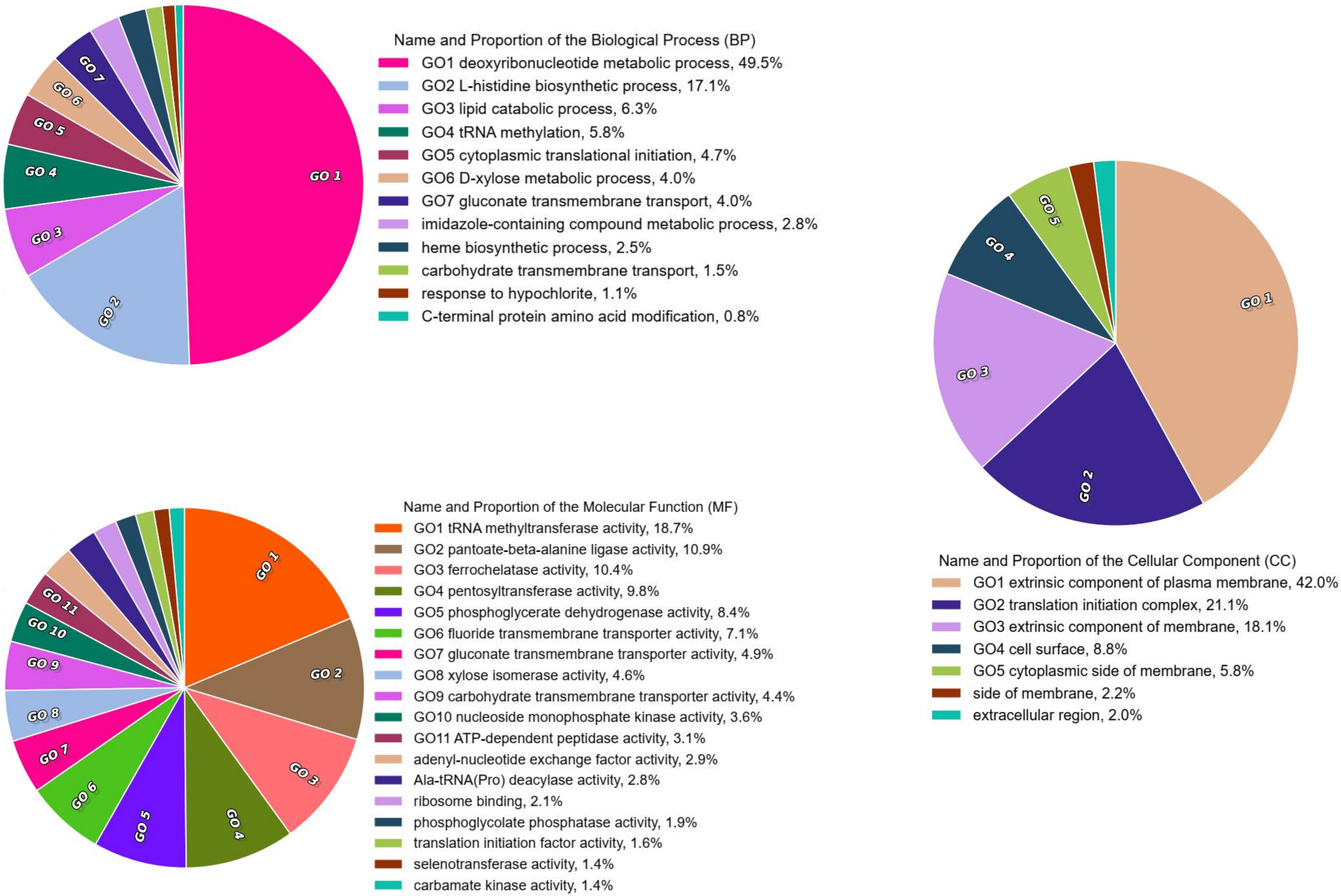
GO 1
GO 2
GO 3
GO 4
GO 5
GO 6
GO 7
Name and Proportion of the Biological Process (BP)
GO1 deoxyribonucleotide metabolic process, 49.5%
GO2 L-histidine biosynthetic process, 17.1%
GO3 lipid catabolic process, 6.3%
GO4 tRNA methylation, 5.8%
GO5 cytoplasmic translational initiation, 4.7%
GO6 D-xylose metabolic process, 4.0%
GO7 gluconate transmembrane transport, 4.0%
imidazole-containing compound metabolic process, 2.8%
heme biosynthetic process, 2.5%
carbohydrate transmembrane transport, 1.5%
response to hypochlorite, 1.1%
C-terminal protein amino acid modification, 0.8%
GO 1
GO 2
GO 3
GO 4
GO 5
GO 6
GO 7
GO 8
GO 9
GO 10
GO 11
Name and Proportion of the Molecular Function (MF)
GO1 tRNA methyltransferase activity, 18.7%
GO2 pantoate-beta-alanine ligase activity, 10.9%
GO3 ferrochelatase activity, 10.4%
GO4 pentosyltransferase activity, 9.8%
GO5 phosphoglycerate dehydrogenase activity, 8.4%
GO6 fluoride transmembrane transporter activity, 7.1%
GO7 gluconate transmembrane transporter activity, 4.9%
GO8 xylose isomerase activity, 4.6%
GO9 carbohydrate transmembrane transporter activity, 4.4%
GO10 nucleoside monophosphate kinase activity, 3.6%
GO11 ATP-dependent peptidase activity, 3.1%
adenyl-nucleotide exchange factor activity, 2.9%
Ala-tRNA(Pro) deacylase activity, 2.8%
ribosome binding, 2.1%
phosphoglycolate phosphatase activity, 1.9%
translation initiation factor activity, 1.6%
selenotransferase activity, 1.4%
carbamate kinase activity, 1.4%
GO 1
GO 2
GO 3
GO 4
GO 5
Name and Proportion of the Cellular Component (CC)
GO1 extrinsic component of plasma membrane, 42.0%
GO2 translation initiation complex, 21.1%
GO3 extrinsic component of membrane, 18.1%
GO4 cell surface, 8.8%
GO5 cytoplasmic side of membrane, 5.8%
side of membrane, 2.2%
extracellular region, 2.0%
